# Supplementary figures and images for: Comparison of interproximal delivery and flow characteristics by dentifrice dilution and application of prepared toothpaste delivery technique
Source: PLoS One. 2022 Oct 17;17(10):e0276227. doi: 10.1371/journal.pone.0276227 (PMC9576051; doi:10.1371/journal.pone.0276227)

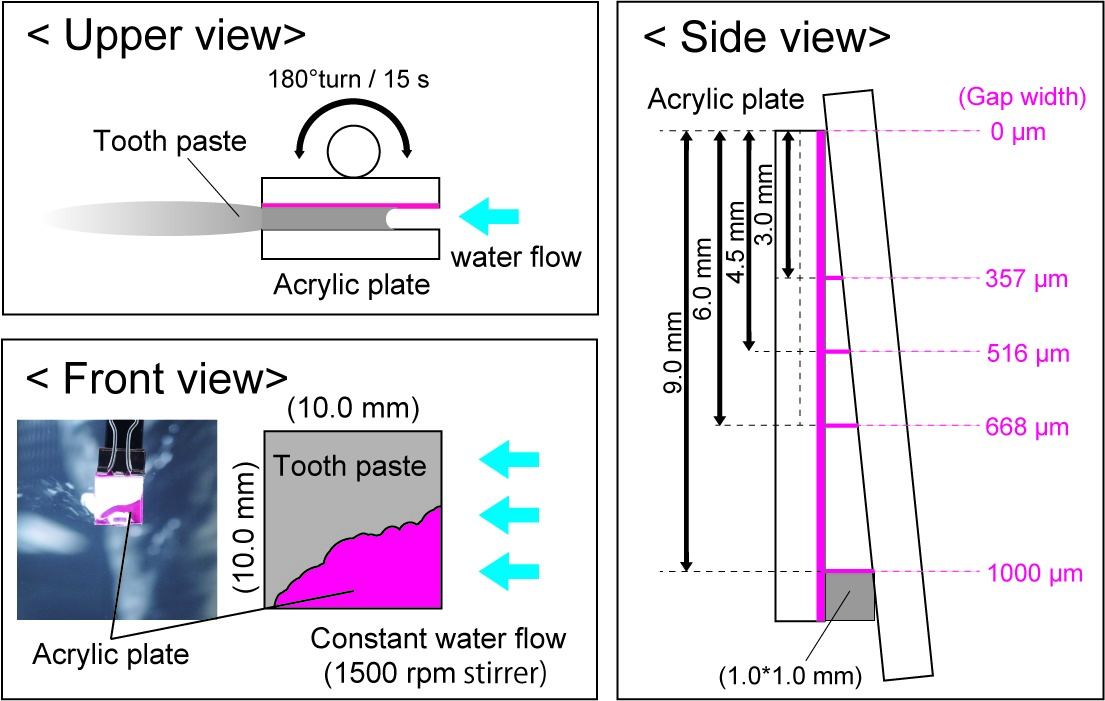

Supplement: S1 Fig — An interdental model is created using two acrylic plates with a 1-mm spacer on one side. The width of the gap changed depending on the distance to the spacer. The gap is approximately 516 μm wide, which is the same as the intraoral tooth spacing at the central 4.5 mm point. The space between the acrylic plates is filled with a dentifrice, and the flow of the dentifrice in a constant stream of water is observed. The model is rotated 180° with respect to the water flow every 15 s; therefore, the amount of dentifrice on the left and right sides is not biased. One side of the acrylic plate is painted red; therefore, the outflow of the dentifrice can be easily observed during the image analysis. (TIF) [file pone.0276227.s001.tif]

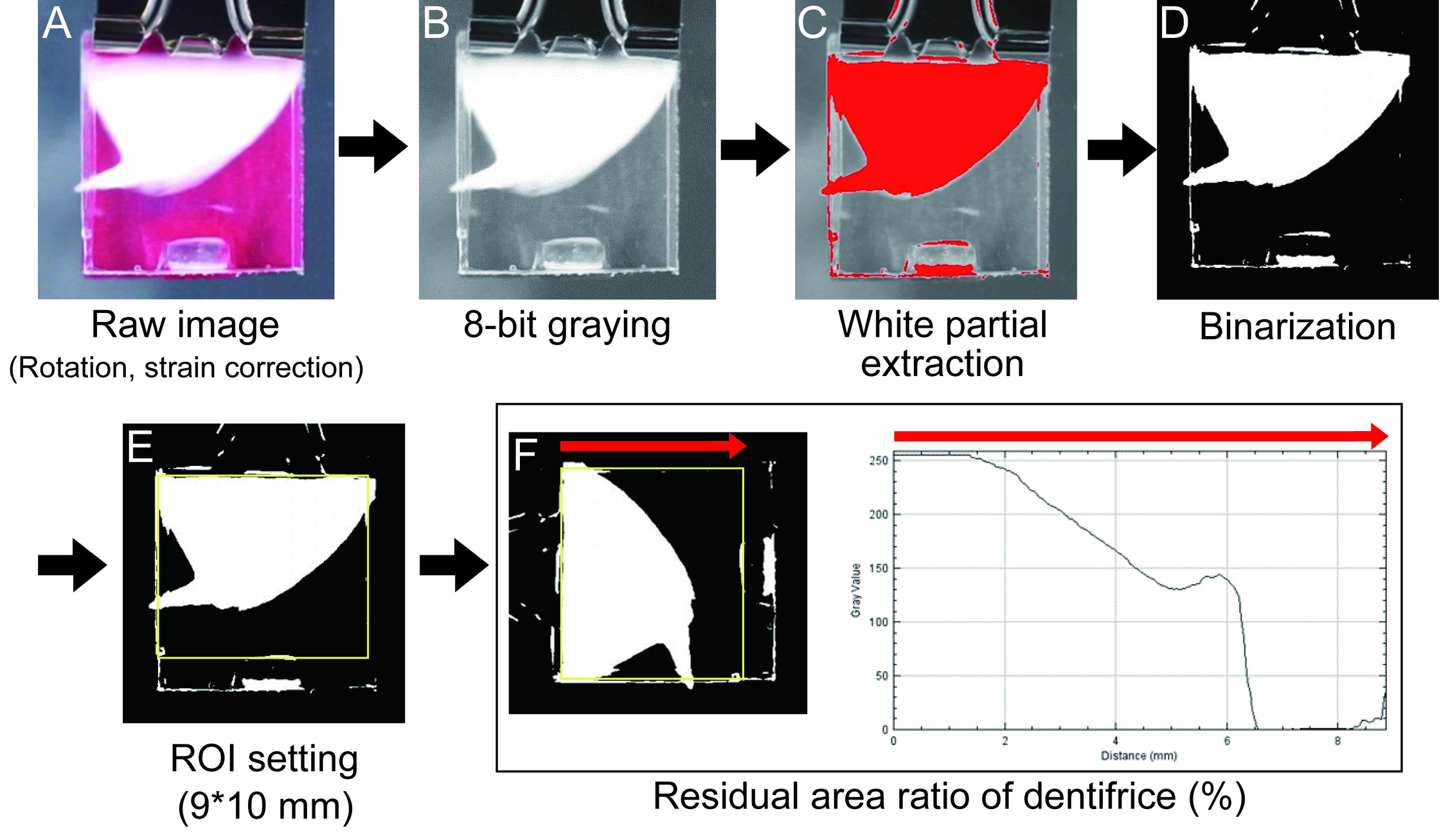

Supplement: S2 Fig — Photographs are captured by a digital camera horizontally 10 cm above the acrylic interproximal model. Images are taken to analyze the amount of dentifrice remaining in the gap. The residual area ratio of the dentifrice (%) is measured using image analysis software (ImageJ, version 1.52). Raw images are converted to eight bits and the white dentifrice portion is measured. The measurement range is 9 mm × 10 mm, excluding the spacer. The signal strength for each pixel is plotted, and the residual area ratio of the dentifrice (%) is calculated. (TIF) [file pone.0276227.s002.tif]

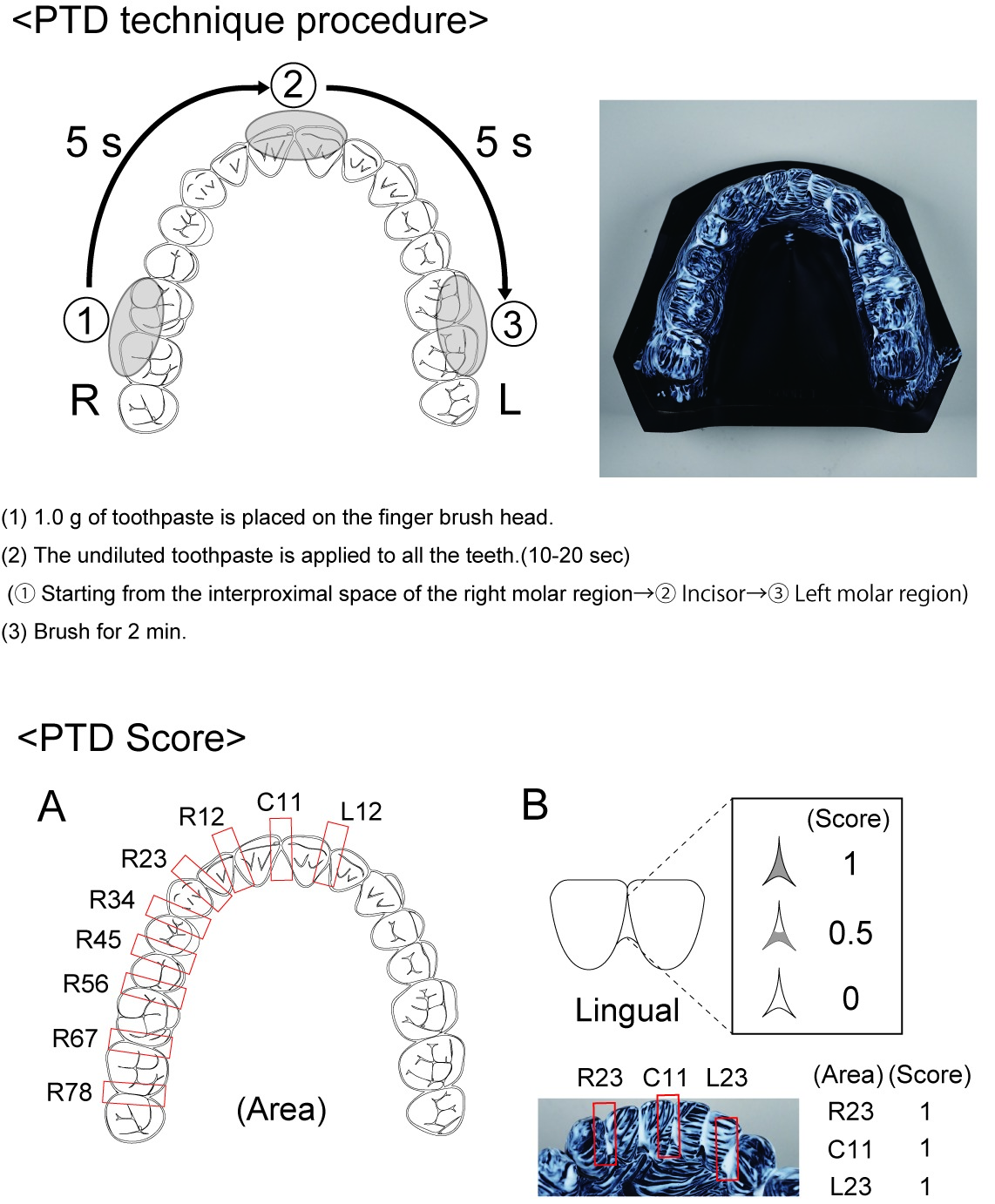

Supplement: S3 Fig — (A) The procedure of the PTD technique is (1) 1.0 g of toothpaste is placed on the brush head, (2) the undiluted toothpaste is applied to all the teeth, starting from the buccal interproximal space of the molar region, (3) the teeth are brushed for 2 min. (B) The PTD score is used to assess the deliverability of the dentifrices in the interproximal area. This score covers only the interproximal area and examines seven points on the right side (R12, R23, R34, R45, R56, R67, and R78), one place in the middle (C11), and seven places on the left side (L12, L23, L34, L45, L56, L67, L78), for a total of 15 places. There are three scores: 0, 0.5, and 1. A score of 1 indicates that the lingual interdental space is filled with a dentifrice. A score of 0.5 indicates half of the space and a score of 0 indicates that the space is completely free of dentifrice. (TIF) [file pone.0276227.s003.tif]
