# Supplementary material for: Comparison of interproximal delivery and flow characteristics by dentifrice dilution and application of prepared toothpaste delivery technique
Source: PLoS One. 2022 Oct 17;17(10):e0276227. doi: 10.1371/journal.pone.0276227 (PMC9576051; doi:10.1371/journal.pone.0276227)
Supplement: Attachment — Submitted filename: Response to Reviewers.docx [file pone.0276227.s004.docx]

Response to Reviewers

Reviewer #1:

I congratulate the authors on a well written manuscript.The material and methods section is well described.The discussion brings out well the highlights of the study.The conclusions are in line with the objectives of the study.All in all a well written manuscript.

> We strongly appreciate the reviewer's comment. We are thankful for the time and energy you expended. We will work hard to make the paper better with this revision.

Reviewer #2:

>Thank you very much for providing important comments. We are thankful for the time and energy you expended. Our responses to the referees’ comments are as follow:

1. LINE 23 - HOW IS THE FLUORIDE TOOTHPASTE COST EFFECTIVE ANY STATSISTICS?

> We appreciate the reviewer's comment on this point. In accordance with the reviewer's comment, we have changed this and added references [2] to following sentence:

Page 5, Line 42-43

Additionally, the application of fluoride-containing dentifrice is cost-effective by a result of the analysis with DMFT index as the outcome [2].

2. LINE 230 - ANY STANDARDISATION OF TIME OF BRUSHING IN ALL THE SUBJECTS?

>Thank you for providing comments. In this experiment, the brushing time is set to 2 minutes. The operators, dentists, trained and calibrated their methods prior to the experiment. The rationale for the 2-minute period is that clinical guidelines in the U.S. and other countries require 2 minutes for brushing instruction.

Page 7, Line 81-82

The operators had sufficient experience in the prepared toothpaste delivery (PTD) technique. The brushing methods were calibrated between the operators.

3. ANY STANDARDISATION OF INTERPROXIMAL DISTANCE OR EMBRASSURE SPACE FOR THE STUDY?

> In our previous study, we conducted experiments using a model with uniform INTERPROXIMAL DISTANCE and published a paper (Ref.12). In this study, we created a black model to clarify the behavior of toothpaste using a model that reflects the arch and tooth morphology in the oral cavity.

12. Satou R, Suzuki S, Takayanagi A, Yamagishi A, Sugihara N. Modified toothpaste application using prepared toothpaste delivering technique increases interproximal fluoride toothpaste delivery. Clin Exp Dent Res. 2020;6(2):188–96.
